# Sex-associated protective effect of early bisphenol-A exposure during enteric infection with *Trichinella spiralis* in mice

**DOI:** 10.1371/journal.pone.0218198

**Published:** 2019-07-10

**Authors:** Karen Elizabeth Nava-Castro, Helena Solleiro-Villavicencio, Víctor Hugo del Río-Araiza, Mariana Segovia-Mendoza, Armando Pérez-Torres, Jorge Morales-Montor

**Affiliations:** 1 Laboratorio de Genotoxicología y Medicina Ambientales, Departamento de Ciencias Ambientales, Centro de Ciencias de la Atmósfera, Universidad Nacional Autónoma de México, Ciudad de México, México; 2 Departamento de Fisiología, Facultad de Medicina Veterinaria y Zootecnia, Universidad Nacional Autónoma de México, Ciudad de México, México; 3 Departamento de Parasitologia, Facultad de Medicina, Universidad Nacional Autónoma de México, Ciudad de México, México; 4 Departamento de Inmunología, Instituto de Investigaciones Biomédicas, Universidad Nacional Autónoma de México, Ciudad de México, México; 5 Departamento de Biología Celular y Tisular, Facultad de Medicina, Universidad Nacional Autónoma de México, Ciudad de México, México; California Pacific Medical Center, UNITED STATES

## Abstract

Bisphenol A (BPA) is an endocrine disruptor compound with estrogenic activity, possessing affinity for both nuclear (ERα and ERβ) and membrane estrogen receptors. The main source of BPA exposure comes from the contamination of food and water by plastic storage containers or disposable bottles, among others, in which case BPA is easily ingested. Exposure to BPA during early pregnancy leads to lifelong effects; however, its effect on the immune system has not been fully studied. Since endocrine and immune systems interact in a bidirectional manner, the disruption of the former may cause permanent alterations of the latter, thus affecting a future anti-parasitic response. In this study, neonate BALB/c mice were exposed to a single dose of BPA (250 μg/kg); once sexual maturity was reached, they were orally infected with *Trichinella spiralis (T*. *spiralis)*. The analyses performed after 5 days of infection revealed a decreased parasitic load in the duodenum of mice in the BPA-treated group. Flow cytometry analyses also revealed changes in the immune cell subpopulations of the infected animals when compared to the BPA-treated group. RT-PCR analyses of duodenum samples showed an increased expression of TNF-α, IFN-γ, IL-4, IL-5, and IL-9 in the BPA-treated group. These findings show a new aspect whereby early-life exposure to BPA contributes to the protection against *T*. *spiralis* by modulating the anti-parasitic immune response.

## Introduction

Endocrine disrupting compounds (EDC’s) are highly lipophilic exogenous substances that bind to hormone receptors. They are capable of interfering in the biosynthesis, storage, metabolism and function of various hormones in the exposed organisms as well as in their offspring [1]. BPA is an estrogenic EDC that binds to different estrogen receptors located in both the nucleus (ERα and ERβ) and the membrane of the cell [2, 3]. This compound is mostly used in the plastic industry with an approximate production of 5x10^9^ kg per year [4], and it is the main component in the manufacture of plastic containers and epoxy resins. Therefore, humans are exposed to BPA on a daily basis, with the average diet (food and beverages contaminated by their plastic containers) being the most important source of risk.

Exposure to BPA becomes even more relevant when it occurs during critical periods of development [5]. In this regard, neonatal and prenatal exposure to this compound can modify the cellular programming provoking several neuroendocrine pathologies during adulthood [6]. For this reason, BPA has been considered as a deleterious compound that seriously affects human health by propitiating a greater susceptibility to diseases such as diabetes mellitus, obesity, and cancer [7, 8].

Regarding the immune system, recent studies have shown that the disruption of the hormonal environment in critical periods of development may induce permanent changes that become apparent before an antigenic challenge during adulthood [9]. The effects of BPA on the reproductive system and even on neuroendocrine function have been extensively studied; however, information about the effects that this compound may induce on the immune system, especially during an antigenic stimulus, is still scarce. Moreover, knowledge concerning BPA, the immune system and its response during trichinosis is very limited. From this point of view, estrogenic EDCs may have similar immuno-regulatory functions to endogenous estrogens. Interestingly, a study conducted by Huimin Yan and colleagues [10] states that prenatal exposure to BPA promotes the production of Th2 cytokines and reduces the percentage of regulatory T cells after infection with *Leishmania major*.

On the other hand, trichinosis is one of the leading zoonotic diseases caused by helminths. In fact, human trichinosis is responsible for financial losses due to severe health problems [11]; further, its incidence is higher in developing countries where the socioeconomic conditions and poor hygiene favor its transmission.

Concerning the immune response against this microorganism, Th2 cells are important in host protective immunity against enteric infection [12]. These cells are characterized by the expression of several cytokines, including IL-4, IL-5, IL-9 and IL-13 [13]. In contrast, T lymphocytes, NK cells, and macrophages play an important role in the initiation, establishment and progression of this disease [14].

Several reports have proven to be controversial, depending on the type of microorganism studied and the effects of BPA during these infections. Elaborating further, a previous report showed that the exposure of perinatal mice to BPA results in a reduced adaptive immune response against Influenza A virus [15]. In another report, mice pretreated subcutaneously with BPA, before and after infection with the *E*. *coli* K12 strain, showed a reduced function and number of innate immune cells [16], thus favoring bacterial colonization. Regarding parasitic infections, several research groups agree that the administration of BPA in animals exerts a protective effect against parasites. For instance, mice infected with *L*. *major* showed an increased expression of Th2 cytokines (IL-4, IL-10 and IL-13) after exposure to BPA during adulthood; on the other hand, animals exposed to BPA during the perinatal stage only overexpressed IL-4 and IFN-γ [10]. The expression pattern of these cytokines was associated with a reduction in the number of Treg cells. Tian and colleagues (2003) [17] reported that adult mice infected with *T*. *spiralis* had a lower parasitic burden after a single dose of BPA prior to infection. However, it is important to highlight that this study did not determine the immune response of the infected mice and were evaluated during a period of chronic infection, in which the parasitic burden is located in muscle; in our study, we determined the effects of an acute infection, which occurs during the infective stage of the parasite. Finally, some groups have explored the direct effects that BPA exerts on different parasites, concluding that BPA impairs the development and survival of the parasites [18–20]. Other studies have proposed that this compound could reduce the life expectancy of infectious parasites through an increased mitochondrial and cytosolic oxidative stress [19].

Based on the above, it is likely that neonatal exposure to BPA could influence the intestinal immune response, thus modifying the susceptibility of the host to *T*. *spiralis* infection during the adult stage. Thus, the aim of this study was to evaluate the effect of neonatal BPA exposure on the intestinal immune response and the susceptibility to acute enteric infection with *T*. *spiralis* in adult mice.

## Methods

### Ethics statement

Animal care and experimental practices were conducted at the Unidad de Modelos Biológicos (UMB) in the Instituto de Investigaciones Biomédicas (IIB), Universidad Nacional Autónoma de México. All experimental procedures were approved by the Institutional Care and Animal Use Committee (CICUAL, permit # 2015–0034) adhering to Mexican regulation (NOM-062-ZOO-1999) and in accordance with the recommendations from the National Institute of Health (NIH) of the United States of America (Guide for the Care and Use of Laboratory Animals). Euthanasia of experimental animals was performed by cervical dislocation after anesthesia with 5% sevofluorane (Abbot, México).

### Animals

BALB/c AnN (H2-d) mice were purchased from Envigo México (Facultad de Química, UNAM, México). The animals were housed at UMB with controlled temperature (22°C) and 12h light-dark cycles, with water and Purina LabDiet 5015 (Purina, St. Louis MO) chow *ad libitum*. After neonatal treatment, both male and female mice were used for experimentation. The animals were checked on a daily basis to make sure they were healthy, clean and with available food and water, as well as with the right light-dark cycle and temperature. Data from 2 independent experiments (5 mice each) are expressed as mean ± standard deviation (SD); N = 10 male mice per treatment (10 control, 10 vehicle and 10 BPA) and N = 10 female mice per treatment (10 control, 10 vehicle and 10 BPA).

### Neonatal BPA exposure

The experimental mice were exposed to BPA at post-natal day (PND) 3 to resemble the human final gestational stage and aiming at the critical developmental window of T lymphocytes. Briefly, 72 h after birth the pups were sexed by ano-genital distance. The pups of both genders received treatment, though whole litters were assigned to experimental groups to avoid pup reallocation stress. The control group received no neonatal treatment. The vehicle group received a dorsal subcutaneous injection of 20 μl corn oil vehicle (Sigma, St. Louis MO). The BPA group received 250 μg/kg bw of BPA. Given that neonate rodents have minimal glucuronidation activity, which is the major metabolic mechanism for BPA clearance [16,19], this dose equals to a brief 5-day exposure according to the FDA reference dose of 50 μg/kg bw/day, performed in a single administration to avoid excessive manipulation stress. Though the main route of exposure is commonly oral, a subcutaneous injection was selected instead as no difference between oral and subcutaneous routes are observed in neonate mice in this case [20]. The pups were weaned at 21 days of age and placed in standard cages, 5 mice per cage.

### Evaluation of endocrine parameters

In order to exclude endocrine factors, we determine three parameters: vaginal opening, estrous cycle and the concentrations of estradiol in serum. From 25 days old forth, the vaginal opening was examined by holding the mice in a dorsal restraint and using a light extension of the peri-vaginal skin. At 8 weeks old, the estrous cycle was determined using a vaginal smear wash of 50 μl saline solution (PiSA, Guadalajara México), followed by Giemsa stain and light microscope observation. Finally, blood samples obtained after sacrifice during the diestrus phase were used to determine serum estradiol levels using the EIA DetectX Serum 17-β-Estradiol kit (Arbor Assays, Ann Arbor MI), following manufacturer’s protocol.

### Experimental groups and Infection procedure

The mice were divided into the following groups after 8 weeks of BPA or vehicle treatment: Control (C), Vehicle (VH), BPA (BPA), Control Infected (C-I), Vehicle Infected (VH-I), and BPA Infected (BPA-I). The infection procedure has been previously described [21, 22]. Briefly, 300 muscle larvae of *T*. *spiralis* suspended in 500μl of 1x PBS were inserted directly into the upper stomach using a gastric catheter. The mice were euthanized after 5 days of infection and the adult worms were recovered by dissecting the small intestine into small sections, washed twice in 1X PBS, and incubated in sterile 1X PBS for 3 h at 37 °C. Following incubation, the sedimented parasites were collected, washed in 1X PBS and quantified under a stereoscopic microscope. All tissue sections were collected immediately after rinsing. To avoid variations due to circadian rhythms, the animals were sacrificed at the same time each day (08:00 am).

### Histological analysis

The duodenum samples were fixed in 4% paraformaldehyde (J.T. Baker, México), dehydrated, and embedded in paraffin. Non-serial, 4μm thick longitudinal tissue sections were cut and mounted on poly-L-lysine coated slides (Sigma, St Louis, MO, USA). The histological analyses were performed with hematoxylin-eosin staining to identify eosinophils and periodic acid Schiff procedure (PAS) for goblet cells. The number of cells from each type in the intestinal glands and villi was calculated using a 40X objective. Several microscope fields, equivalent to a 1 mm^2^ area, were analyzed for each mouse. The empty areas within the tissue were discarded using the software Image J. A total area of 1 mm^2^ of villi and intestinal glands was analyzed per group. The identification criteria were based on the morphological characteristics of the cells.

### Flow cytometry

The duodenum was mechanically disaggregated using a 50 μm nylon mesh and washed with PBS. Erythrocytes were lysed with ACK buffer (150 mM NH_4_Cl, 10 mM KHCO_3_, 0.1 mM Na_2_EDTA, pH 7.3) for 10 min and washed with PBS. The duodenum samples were finely cut and incubated 20 min in digestion medium RPMI 1640, 10 U/mL DNase (Roche, Mannheim Germany), 0.5 mg/mL type IV Collagenase (Sigma, St. Louis MO). Digestion was stopped by adding 50 μL FBS and mesh disaggregation was performed followed by PBS wash. The cells were resuspended in FACS buffer (PBS, 2% FBS, 0.02% NaN_3_).

The lymphocyte subpopulations were characterized using the following antibodies: FITC-conjugated anti-CD3ε (145-2C11), PE-conjugated anti-CD4 (GK1.5), PE-Cy5-conjugated and PE-conjugated anti-CD8 (53–6.7), and CD19+PE. FACS analysis was done using an Attune cytometer (Life Technologies) and the software FlowJo (Treestar Inc.).

### RT-PCR

The duodenal samples were frozen in TRIzol (Ambion, Carlsbad CA) immediately after necropsy. Total RNA was extracted using the same reagent, following manufacturer’s protocol. Briefly, the tissue was disrupted in TRIzol (1 ml/0.1 g tissue) and 0.2 mL of chloroform were added per each ml of reagent. The aqueous phase was recovered after centrifugation at 13,000 rpm for 15 min. The RNA was precipitated with isopropyl alcohol, washed with 75% ethanol, and resuspended in RNAse-free water. RNA concentration was determined by absorbance at 260 nm and its integrity verified by electrophoresis in a 1.0% agarose gel.

Total RNA samples were immediately reverse-transcribed, using M-MLV Reverse Transcriptase (Promega, Madison WI) and dT12–18 primers (Invitrogen, USA). The cDNA was used in semi-quantitative PCR assays using TaqDNA polymerase (Biotecnologías Universitarias, UNAM. México) and the following primers (Table 1). The relative expression of each amplified gene was determined by densitometric analysis, using the 18S-ribosomal RNA amplicon as a housekeeping control.

**Table 1:** Primer sequence. The primers were designed based on reported sequences from the Gene Databank (NCBI, NIH).

Primer		Sequence	Tm (°C)	Length (bp)
IL-2	Sense	TCTACAGCGGAAGCACAG	61	490
	Anti-sense	TCATCGAATTGGCACTCA		
IL-4	Sense	TCATGGGATGATGATGATAACCTGCT	62	502
	Anti-sense	CCCATACTTTAGGAAGACACGGATT		
IL-5	Sense	CATCGAACTCTGCTGATAGCC	65	500
	Anti-sense	TCTCCGTCTTTCTTCTCCACA		
IL-10	Sense	AACTGGTAGAAGTGATGCCCCAGGCA	63	237
	Anti-sense	CTATGCAGTTGATGAAGATGTCAAA		
IL-12	Sense	CCAGGTGTCTTAGCCAGTC	60	450
	Anti-sense	CTCGTTCTTGTGTAGTTCCAG		
TNF-α	Sense	GGCAGGTCTACTTTGGAGTCATTGC	63	300
	Anti-sense	ACATTCGAGGCTCCAGTGAATTCGG		
IFN-γ	Sense	AGCGGCTGACTGAACTCAGATTGTAG	60	247
	Anti-sense	GTCACAGTTTTCAGCTGTATAGGG		
TGF-β	Sense	CTTCAGCTCCACAGAGAAGAACTGA	61	298
	Anti-sense	CACAATCATGTTGGACAACTGCTCC		
18S	Sense	CGCGGTTCTATTTTGTTGGT	60	219
	Anti-sense	AGTCGGCATCGTTTATGGTC		

### Anti-trichinella IgG level in serum

The analyses of total anti-*T*. *spiralis* IgG levels in serum were performed on 96-well plates (flat bottom). The plates were previously sensitized with a dilution of secreted *T*. *spiralis* antigen (20 ng of protein), followed by the serum of infected animals from each group (1:1,000), washed and blocked with a 1% albumin solution. Afterward, HRP conjugated α-IgG mice antibodies were incubated for 2h, washed, and TMB was added to the wells, the reaction was stopped and the plates were analyzed in a Stat Fax 4200 microplate reader at 492 nm (Awarness Technology).

### Statistical analysis

The experimental design considers 3 independent variables: sex (two levels: male or female), neonatal exposure (Control, Vehicle or BPA) and infection (I). Data from 2–3 independent experiments were analyzed with the Prism 6 software (GraphPad Software Inc.) and charted as mean ± standard deviation. Data distribution normality was determined with a Shapiro-Wilk test. Thereafter, a one-way ANOVA (α = 0.05) was performed followed by a Tukey *post-hoc* test (*P* < 0.05).

## Results

### Endocrine parameters

Since BPA is an endocrine disruptor and infections are important factors altering the endocrine parameters of the host, it is important to discriminate whether its effects are due to the direct or indirect effects of neonatal endocrine disruption on the immune system or if it is caused by persistent hormonal alterations in the host. To determine if a single dose of BPA (250 μg/kg bw) has any reproductive effects, the vaginal opening of female mice was used as an indicator of puberty onset. Contrary to other works, where higher or prolonged neonatal BPA exposure resulted in a modified puberty onset, in our scheme this compound did not alter it (Table 2). In addition, the estrous cycle was also monitored once sexual maturity was fully established (8 weeks old), finding no significant difference between the animal groups. Furthermore, its exposure had no effect on baseline estradiol levels in serum during the diestrus phase (Table 2). In males, no behavioral, morphological or hormonal alterations were found.

**Table 2:** Assessment of endocrine parameters in female mice, using the vaginal opening as an indicator of puberty onset.

Group	Vaginal Opening (days)	Serum 17-β estradiol [pg/ml]	Completed Estrous cycle
Control	30	45 ± 5	Yes
VH	30	48 ± 2	Yes
BPA	30	49 ± 3	Yes
Control—Infected	30	47 ± 3	Yes
VH—Infected	30	46 ± 6	Yes
BPA—Infected	30	48 ± 3	Yes

The serum levels of 17-β-estradiol at diestrus phase (~12 weeks old) were measured by EIA. Data from 2 independent experiments (5 mice each) are expressed as mean ± SD; N = 10 male mice by treatment (10 control, 10 vehicle and 10 BPA) and N = 10 female mice by treatment (10 control, 10 vehicle and 10 BPA).

### Analysis of intestinal parasitic burden

**Fig 1:**
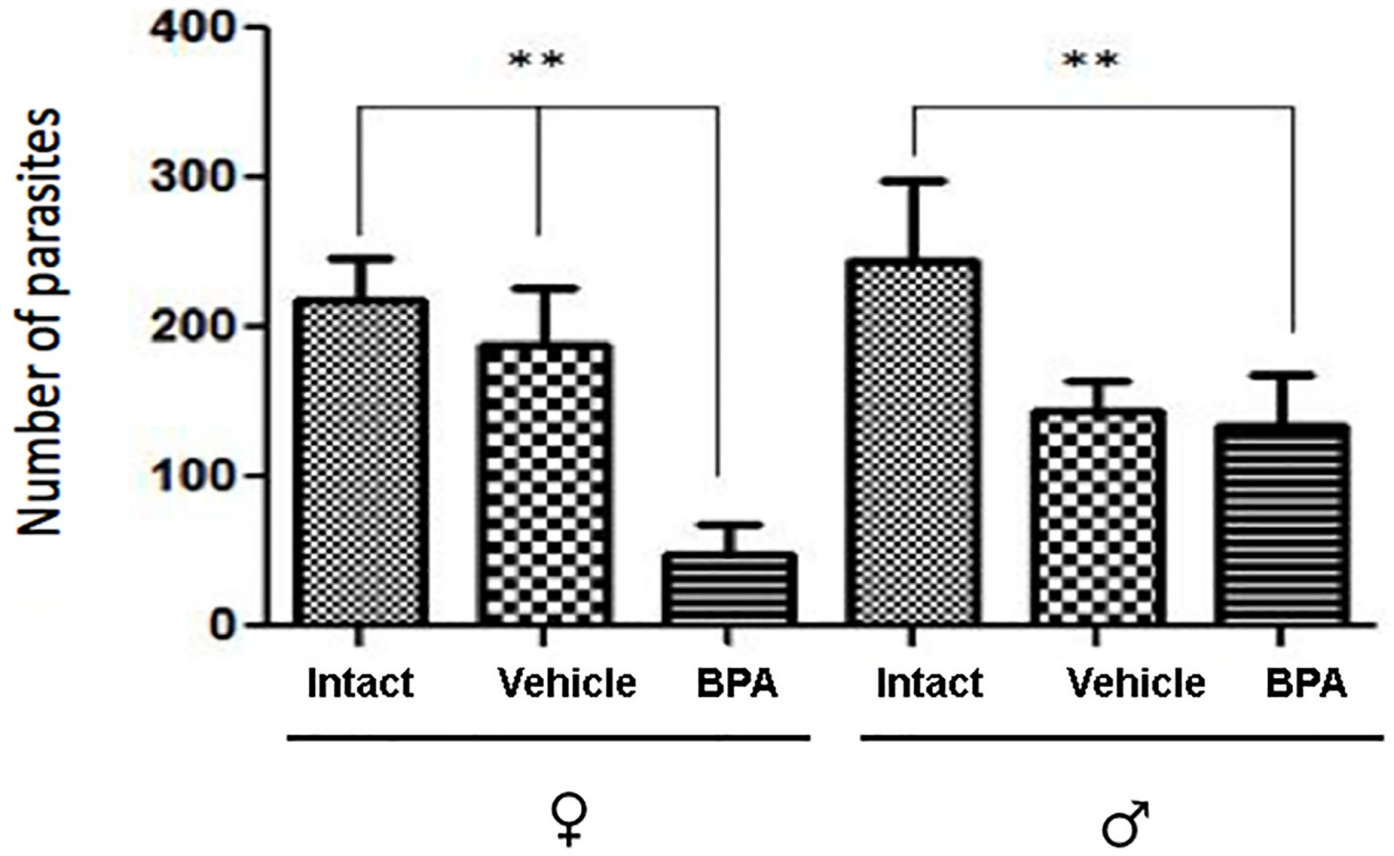
Parasitic burden in the small intestine. Treatment with BPA significantly decreased the number of parasites in male and female mice. The animals were infected with *T*. *spiralis* larvae and sacrificed after 5 days. Bars represent female and male mice from the three infected groups: control infected (C-I), vehicle infected (VH-I) and BPA infected (BPA-I). Data are shown as mean ± standard deviation. *** *P*<0.05.

Regarding the infected animals, we observed a significantly decreased parasitic burden in the small intestine of female mice previously treated with BPA (BPA-I) in comparison with the control and vehicle (VH) groups (Fig 1). This effect was not observed in the male BPA-I group. Interestingly, the reduced parasitic burden was related to sex in such a way that the females treated with BPA had a lower number of larvae than their male counterparts, representing a significant BPA-induced protection in females (Fig 1).

**Fig 2:**
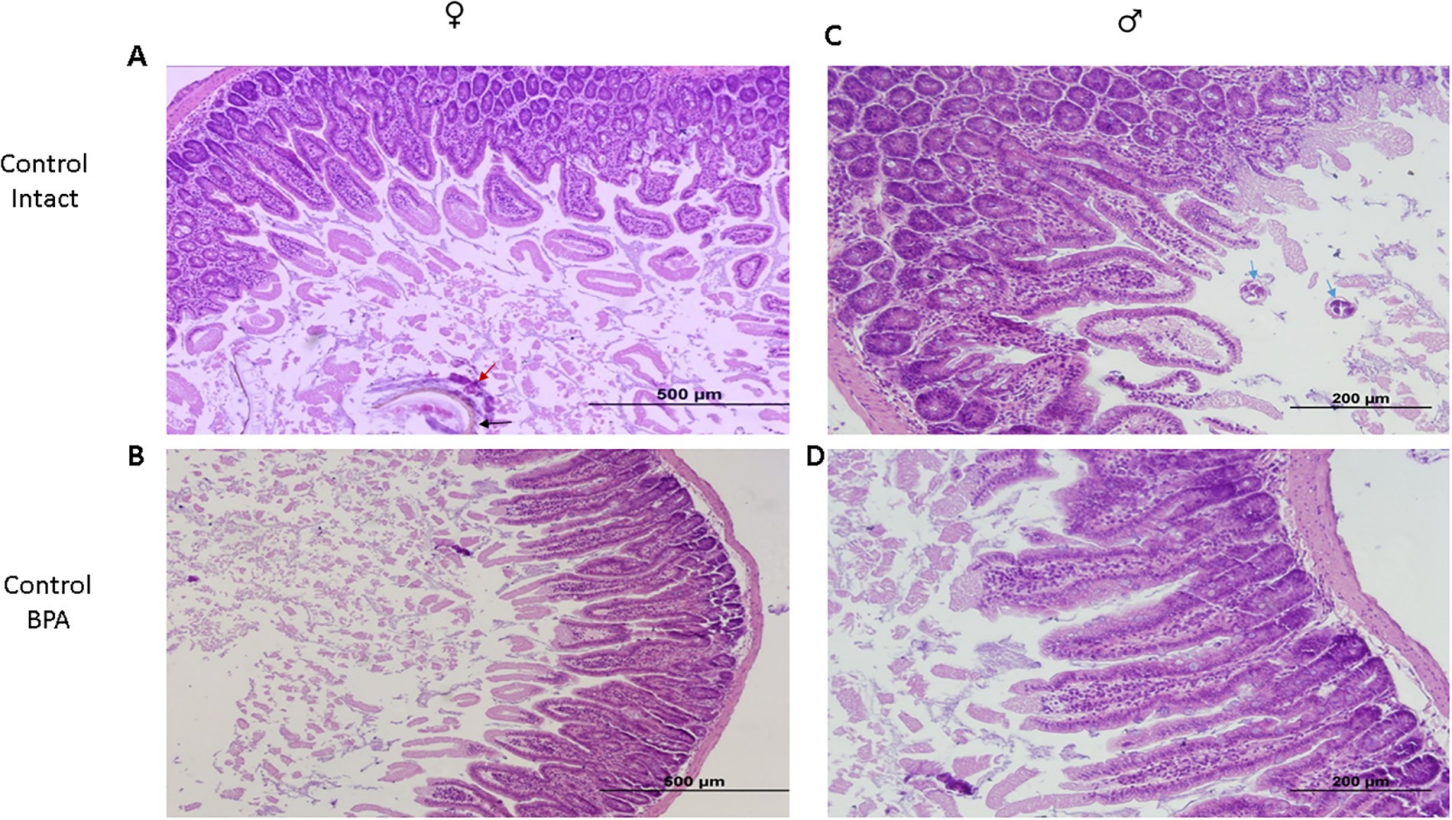
Histological evaluation of the duodenal inflammatory infiltrate associated with *T*. *spiralis* infection. Photomicrograph of longitudinal sections of the proximal duodenum of male and female control infected (C-I) mice (a, c, respectively), and male and female BPA infected (BPA-I) mice (b, d, respectively). The black arrow indicates *T*. *spiralis* larvae, red arrow indicates eosinophils attached to the parasite, and blue arrows indicate encysted larvae.

The difference in parasitic burden, as related to sex or treatment, is based on the histological evaluation of duodenum sections from the studied mice. The histological samples from both sex groups were stained with H&E and analyzed under the microscope, revealing the morphological changes associated with *T*. *spiralis* infection, such as widening of the villi, loss of apical epithelial integrity and inflammatory infiltrate at the lamina propria (Fig 2). These analyses showed that male mice from the C-I group had a greater number of encysted larvae and lower leukocyte infiltration than their female counterparts. We also observed that mice from the BPA-I group had a higher number of goblet cells distributed in the villi and intestinal gland epithelium in both sexes.

**Fig 3:**
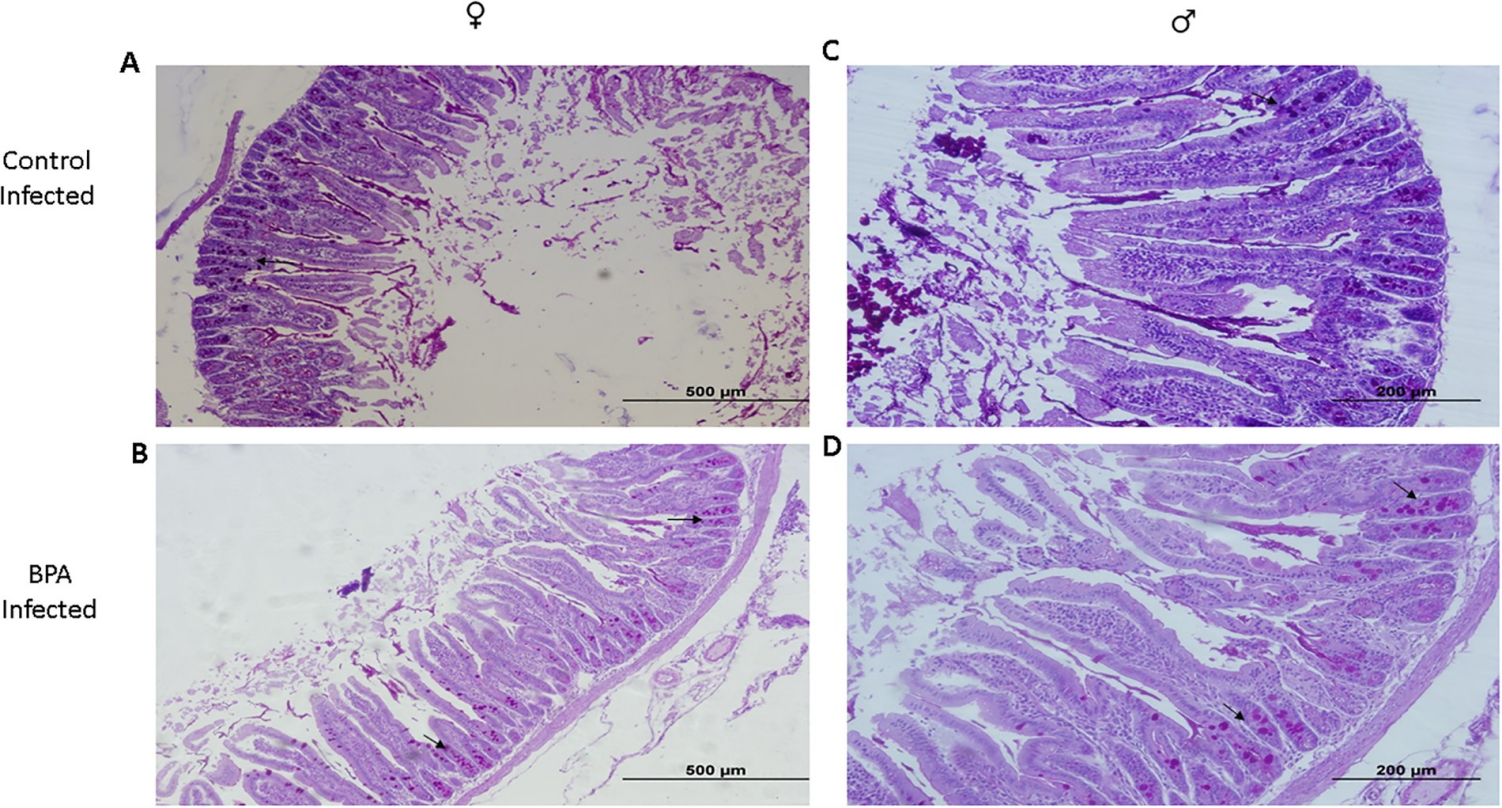
Histological analysis of the duodenal inflammatory infiltrate associated with *T*. *spiralis* infection. Photomicrograph of longitudinal sections of proximal duodenum of male and female control infected (C-I) mice (a, c, respectively), and male and female BPA infected (BPA-I) mice (b, d, respectively). Goblet cells (arrow) are stained red to purple.

In contrast, these cells were exclusively confined to the intestinal gland epithelium in the C-I group, as determined with the periodic acid Schiff staining (PAS) (Fig 3). Interestingly, both mice groups showed an increased mucus discharge towards the intestinal lumen, which may be associated with an altered duodenum function.

### Phenotypic characterization of leukocytic infiltrate in the duodenum

**Fig 4:**
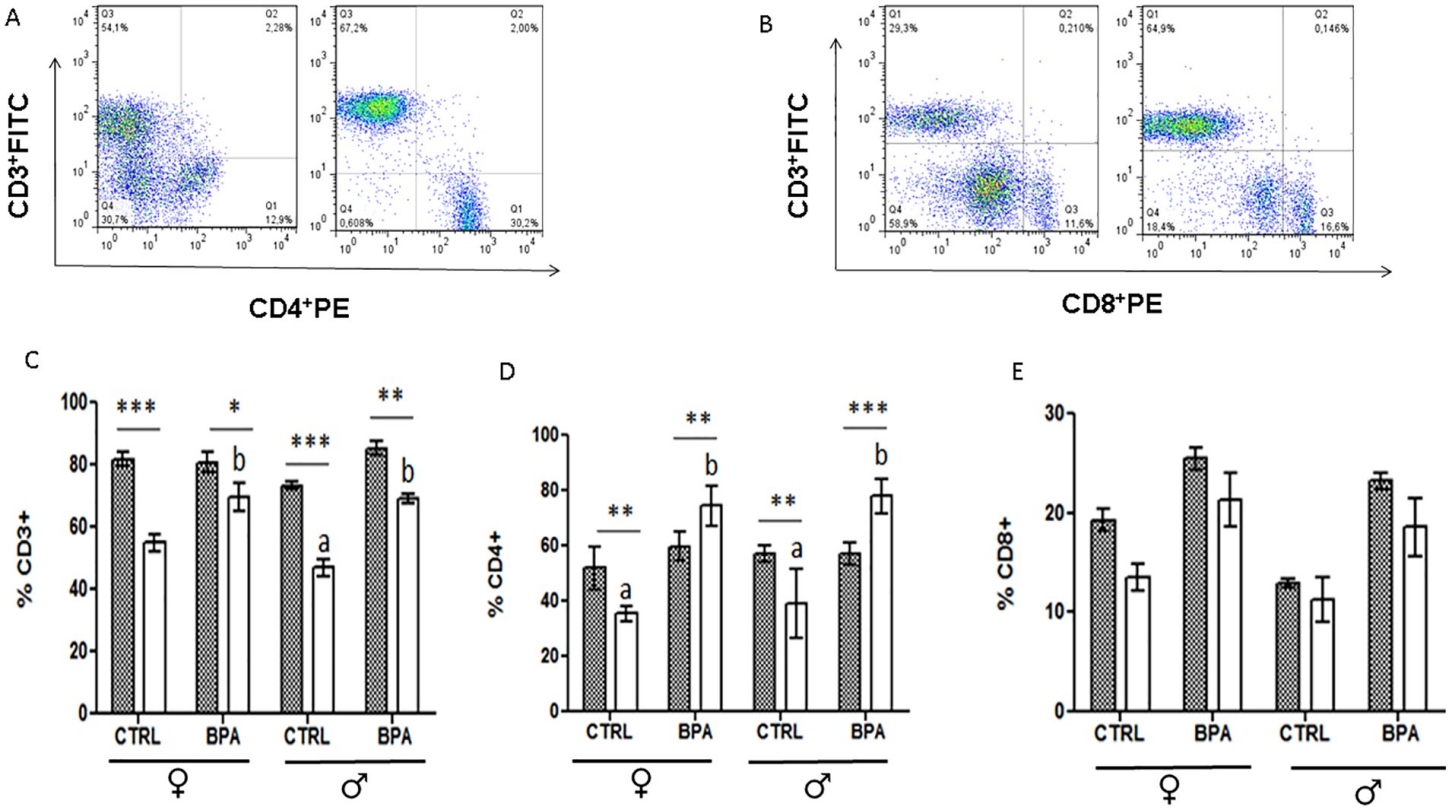
Analysis of the inflammatory infiltrate in the mesenteric lymph nodes. Analysis of immune subpopulations frequencies by flow cytometry, (**a**) and (**b**) are representative dot-plots of cytometric analysis algorithm of T cells (CD3^+^), and CD4^+^ or CD8^+^cells, respectively. The graphs represent the frequencies of (**c**) T cells, (**d**) CD4^+^T cells, (**e**) CD8^+^ T cells. Literals-ANOVA 1way/Tukey between groups *P*<0.05 Asterisks- ANOVA 2 way/Bonferroni * *P*<0.05, ** *P*<0.01, *** *P*<0.001.

Phenotypic characterization of the T cell subpopulations present in the duodenum was performed by flow cytometry (Fig 4a and 4b). There were no significant differences between the non-infected groups concerning the percentage of these cells; however, this percentage was decreased significantly in the infected groups regardless of treatment (Fig 4c).

The control groups showed a decreased percentage of Th cells after infection with *T*. *spiralis* (Fig 4d). In contrast, the BPA-treated groups showed a significantly increased frequency of Th cells during the infection in comparison with the II group. On the other hand, the percentage of TCD8^+^ cells was decreased in all of the infected groups (Fig 4e). In similar manner, we found that the mice treated with BPA had a higher frequency of these cells than the C-I group. Further, female mice had a higher percentage of cytotoxic lymphocytes than their male counterparts, suggesting this increment could be one of the mechanisms through which BPA is able to reduce the parasitic load.

### Cytokine expression pattern

The immuno-modulatory effects of BPA over the cytokine expression profile in the duodenum of infected mice were clearly dimorphic, *i*.*e*. female mice showed a higher cytokine expression than males (Table 3). The expression of Th1 cytokines (IL-2 and IL-12) seemed unaffected by BPA in the infected group; however, the expression of Th2-related cytokines (IL-4 and IL-5) was increased ~8-fold. Although these effects were similar in both sexes, the female BPA-I group showed a higher expression of Th2 cytokines than the corresponding male group. A similar effect was observed in the expression of the proinflammatory cytokines IL-6 and TNF-α, which were increased by 5- and 9-fold, respectively (Table 3). The cytokine expression profile of the duodenal mucosa from BPA-treated and untreated mice was of particular interest because this tissue is in direct contact with the parasites.

**Table 3:** Effects of BPA exposure over the cytokine expression pattern in the duodenum of mice infected with *Trichinella spiralis*.

Cytoline Family	Cytokine	Gender	Groups		
			Control—Infected	VH—Infected	BPA—Infected
Th1	IL-2	♀	9.3 ± 2.5	10.9 ± 1.8	8.9 ± 1.6
		♂	5.1 ± 1.9	5.0 ± 0.9	6.1 ± 0.5
	IL-12	♀	7.9 ± 1.3	7.4 ± 0.9	7.5 ± 2.1
		♂	5.0 ± 0.8	4.9 ± 1.1	5.2 ± 1.1
Th2	IL-4	♀	15.4 ± 2.4	15.0 ± 1.3	28.6 ± 4.5 **
		♂	10.2 ± 1.8	9.89 ± 10.8	20.4 ± 2.5 **
	IL-5	♀	14.6 ± 0.9	14.0 ± 1.2	30.5 ± 3.9**
		♂	8.9± 1.1	10.0 ± 0.9	21.0 ± 2.8**
Pro-inflammatory	IFN-γ	♀	12.1± 0.6	11.9 ± 0.5	23.9 ± 1.9**
		♂	6.3 ± 0.5	6.0 ± 1.1	18.6 ± 3.5**
	TNF-α	♀	11.6 ± 0.8	11.0 ± 1.2	27.4 ± 4.2**
		♂	10.9 ± 1.0	10.0 ± 0.9	23.6 ± 3.3**
Regulatory	IL-10	♀	22.4 ± 0.9	21.9 ± 1.1	36.3 ± 3.9**
		♂	14.8 ± 0.4	14.6 ± 0.5	28.6 ±2.9**
	TGF-β	♀	10.6 ± 1.2	11.0 ± 1.9	19.2 ± 4.5**
		♂	6.3 ± 0.7	6.4 ± 1.0	10.7 ± 4.2**

Relative expression (RE), [Optical Density (OD) cytokine /OD housekeeping gene (18s)]. Data are presented as RE ± SD.

*P<0.05,

**P<0.01.

n = 10 (5 mice in two different experiments). ♀: females. ♂ Males.

### Humoral immune response

**Fig 5:**
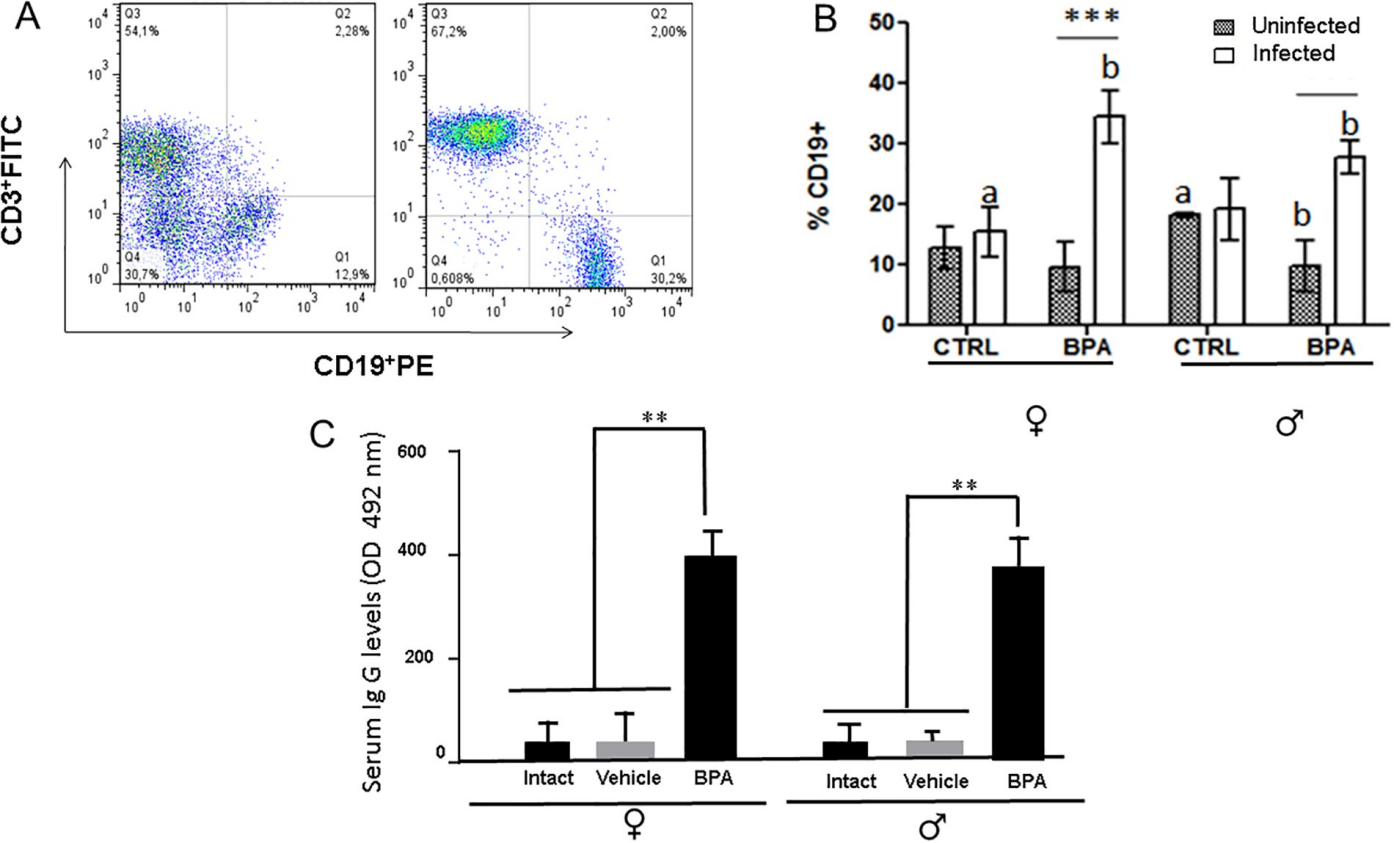
Analysis of B cell subpopulations frequencies by flow cytometry, (**a**) Representative dot-plot of cytometric analysis algorithm of B cells (CD3^-^CD19^+^). The graph represents the frequencies of B cells **(b)**. The levels of anti *T*. *spiralis* antibodies are depicted in (**c**). Literals-ANOVA 1 way/Tukey between groups *P*<0.05 Asterisks- ANOVA 2 way/Bonferroni * *P*<0.05, ** *P*<0.01, *** *P*<0.001.

Fig 5 depicts the gating selection of B cell subpopulations in the mesenteric lymph nodes as well as their relative number (%) in these organs (Fig 5a and 5b, respectively). In addition, the serum levels of anti-*T*. *spiralis* IgG antibodies in the BPA-I groups are shown in Fig 5c. Interestingly, BPA exposure increased the number of B lymphocytes in in the infected groups, being higher in females than in male mice. Further, B cell activity was also impacted in these groups, as shown by the ~3 fold increased levels of anti-*T*. *spiralis* IgG antibodies in both sexes (Fig 5c).

## Discussion

The aim of the present study was to evaluate the effects of a single neonatal administration of BPA in mice and its effects on the establishment of the gastrointestinal parasite *T*. *spiralis* and the corresponding duodenal immune response.

Since BPA is widely used as a monomer in the production of polycarbonate plastics, epoxy resins, and dental sealants [1], this compound can be released from these materials due to high temperatures, acidic conditions or enzymatic processes. Thus, the main source exposure to BPA in animals and humans is through food and beverages contaminated by their plastic containers, from which it can be released and ingested [23]. The results of previous BPA studies are controversial; regardless, most have deemed it as a hazardous material. However, since these studies differ widely (BPA concentration, *in vivo* animal model, *in vitro* tests, *ex vivo* in human cells, parasite life stage, antigenic challenge, or sex), there is no clear pattern regarding its effects on the immune system. Therefore, more studies are needed to elucidate the mechanisms involved in these effects.

Different BPA effects have been reported on the immune system cells; however, they vary depending on whether they were performed as *in vivo* or *in vitro* models, the animal species used, the dose, the administration route and the animal’s development stage in which BPA is administered. Furthermore, many reports do not consider that the immune response must be studied under some antigenic challenge, so there is little information about the BPA effects on the immune system during an infectious process. For instance, in general, BPA has been shown to alter the macrophages and dendritic cell cytokine secretion, their activation phenotype and their adhesion molecules expression. BPA modulate endogenous steroid responses and immune cell functions. Although most studies focus on their reproductive effects, their potential effects on immune cells and even more, on the immune response towards pathogens, should draw attention, given the expression of hormonal receptors by immune cells. Despide the fact that a lot of studies have evaluated the BPA effect with different variables such as BPA concentration, type of model, administered BPA dose, life stage or antigenic challenge used, one common element remains: BPA can differentially modulate the immune response. However, more studies are needed with the aim to elucidate the possible mechanisms by which this takes place.

In the present study, we evaluated the endocrine alterations produced by a previously tested BPA exposure scheme, *i*.*e*. single 250 μg/kg bw dose at postnatal day 3 in mice. In our results, we did not observe any hormone alterations regarding the puberty onset, regularity of the estrous cycle, or changes in the basal estradiol serum levels in female mice; neither were any effects observed in male mice. It is important to mention that we observed significantly decreased parasitic loads, which could be strongly related to the immunological function rather than the hormonal background. Previous reports concerning the function and modulation of the immune response have not considered specific antigenic challenges, thus, there is little evidence about the effects of BPA in the context of an infectious disease, specifically on parasitic infections. In this sense, this study provides original scientific evidence regarding the effect of neonatal BPA administration on the intestinal immune response in mice against *T*. *spiralis*.

A previous report on the protective effect of BPA against *T*. *spiralis* [17] did not consider the change in relative number (%) of immune cells in the BPA-treated mice nor the cytokine expression pattern; in addition, it also analyzed the number of total larvae in the muscles of infected animals, which means that the life cycle of the parasite was already complete (chronic infection). In contrast, the present study included the infective stage of the parasite, and analyzed the associated immune response during its acute infection phase. Although the same parasite was used in both studies, the results are not easily comparable. Following this line of thought, the present study showed that only a single dose of BPA administered during a critical developmental stage in mice prevents the intestinal establishment of the parasite through increased number of both cytotoxic immune cells and B lymphocytes. Further, the expression of IL-10 was higher than that of pro-inflammatory cytokines; such increment has also been reported in another *in vitro* model using a human macrophage cell line. These observations open the possibility of studying the effects of BPA on the function of T regulatory cells.

Other studies have shown that the perinatal exposure to BPA increases the susceptibility to infection with *Nippostrongylus brasiliensis* in rats [9]. As for the direct effects of BPA upon the parasites, Tan et al., (2015) reported the reduced life expectancy of *Caenorhabditis elegans* (*C*. *elegans*) induced by this estrogenic compound; also, BPA accelerated its aging process by increasing the mitochondrial and cytosolic oxidative stress, as well as ROS generation [19].

Another striking result of our study was that female mice showed a greater cytokine secretion in the presence of *T*. *spiralis* than males, which could be associated with hormonal differences between the sexes since estrogens can enhance the immune response, an effect that has also been associated with autoimmune diseases.

It must be highlighted that the age at the time of BPA administration is crucial for the development of the immune system, as it has been reported that exposure during critical developmental stages can modify some epigenetic patterns affecting the expression of estrogen and other receptors in reproductive organs, brain, and the immune system [24, 25]. Concerning the latter, the possibility of these epigenetic changes in immune cells is a novel hypothesis that needs to be confirmed.

Finally, EDCs can modulate the response of endogenous steroids and cell functions, although most studies have been focused on their effects in reproduction. However, since immune cells also have hormone receptors, the effect of EDCs on the modulation of the immune response against different pathogens should also be studied. Despite the existence of several studies evaluating the effects of BPA, the use of different variables such as BPA concentration, experimental design, administered BPA dose, life stage, or antigenic challenge used, makes difficult to find a clear pattern regarding the activation of the immune system. Regardless, the shared characteristic between these studies is that BPA can differentially modulate the immune response by increasing or decreasing the number and activity of Th1, Th2, and Th17 cells. However, more studies are needed to elucidate how BPA can modulate the immune response.

## Conclusion

The present results provide further proof that early exposure to BPA affects cytokine expression and antibody production, turning into a Th1 response during the adult life and resulting in a decreased susceptibility to intestinal parasitic infection with *T*. *spiralis*. These effects emphasize the high sensitivity of the immature immune system to BPA during the neonatal period, altering its effector mechanisms in adult life. Due to its ability to modulate the cytokine expression profile and the constitution of several immune cell populations, the administration of BPA during critical periods of development could serve as a preventive tool against infections with *T*. *spiralis* in the adult stage.

## Supporting information

S1 Checklist ARRIVE guidelines checklist. (PDF)
